# Multi-Channel Real-Time Condition Monitoring System Based on Wideband Vibration Analysis of Motor Shafts Using SAW RFID Tags Coupled with Sensors

**DOI:** 10.3390/s19245398

**Published:** 2019-12-07

**Authors:** Pau Caldero, Dominik Zoeke

**Affiliations:** 1 Institute of Microwaves and Photonics (LHFT), Friedrich-Alexander University of Erlangen-Nuremberg, Cauerstraße 9, 91058 Erlangen, Germany; 2 Siemens Mobility, Otto-Hahn-Ring 6, 81739 Munich, Germany

**Keywords:** SAW, augmented RFID, IIOT, condition monitoring

## Abstract

While there is a wide range of approaches to monitor industrial machinery through their static components, rotating components are usually harder to monitor, since sensors are difficult to be mounted on them and continuously read during operation. However, the characteristics of rotating components may provide useful information about the machine condition to be included in monitoring algorithms, specially for long-term data analysis. In this work, wireless vibration monitoring of rotating machine parts is investigated using surface acoustic wave (SAW) radio frequency identification (RFID) tags coupled with sensors. The proposed augmented transponder solution, combined with low-latency interrogation and signal processing, enables real-time identification and wideband vibration sensing. On top of that, a multi-channel interrogation approach is used to compensate motion effects. This approach enhances the signal-to-noise ratio of low-power high-frequency components present on the vibration signatures and enables discriminant information extraction from rotating machine parts. Final feasibility is evaluated with induction motors and vibration measurements on rotating shafts are verified. In addition, a condition classification algorithm is implemented in an experimental setup based on different motor states. The results of this work open the possibility to feed predictive maintenance algorithms using new features extracted in real-time from wideband vibration measurements on rotating components.

## 1. Introduction

In the industrial internet-of-things (IIOT) field, condition monitoring and fault diagnosis of rotating machinery are very valuable topics investigated to improve maintenance and scheduling efficiency, recognize faulty machines and avoid consequent economic losses caused by unexpected shutdowns. Besides, factories with a mature level of automation demand a combination of sensor technologies which, in turn, must be integrated in a compatible framework, eventually involving fusion of data from different sources. In the meantime, this heterogeneous information must be extracted, combined and managed. To enable efficient real-time communication between devices and automatic control applications, low-latency data acquisition, processing and transmission are desired characteristics for potential system solutions.

### 1.1. Vibration Monitoring

A major focus in Industry 4.0 is on engines, which constitute the backbone of production lines. Continuous monitoring of vibration allows preventive tasks, such as premature detection of bearing damages or shaft misalignment, and the possibility to respond efficiently according to the diagnosis. In particular, condition monitoring and, with it, the feasibility of scheduling maintenance processes in advance (predictive maintenance) come to the foreground, minimizing asset losses and costs while maximizing production efficiency.

Vibration monitoring of rotating machine parts is targeted in this work. Continuous sensor data transmission during device under test (DUT) operation enables early detection of potential structural failures and the corresponding adoption of mitigating actions before an accident occurs. Wired interfaces to directly connect sensors and acquire data continuously are usually robust, while data transmission reach minimum latencies; however, they can be hardly mounted in a reasonable, maintenance-free way on rotating parts. In contrast, using sensors in combination with standard wireless interfaces typically run into issues with communication delays or lower measurement rates, while usually running on battery-powered solutions, what causes a reduced performance for real-time applications.

### 1.2. Solution Proposed

Surface acoustic wave (SAW) radio frequency identification (RFID) technology offers a wireless, passive solution, which can be augmented by coupling with commercial off-the-shelf (COTS) sensors without special manufacturing requirements [1,2]. Thus, a system based on SAW RFID coupled with external sensors is proposed. The system addressed in this work aims at vibration measurement involving rotating or, at least, moving machine parts which are a burden with regards to cabling and measurement update rates. Using a direct sensing principle instead, sensors can be attached even to fast rotating parts, such that vibration information is acquired right at the spot of interest. Furthermore, an aimed characteristic for the system is to achieve fast update rates, which enable high-frequency vibration signal component acquisition using a robust transponder solution with no computation unit or battery, i.e., resistant to harsh environments. On top of that, a multi-channel interrogation principle is implemented to compensate for the antenna anisotropic radiation pattern effects. This principle enhances the signal-to-noise ratio and, thus, the contribution of low-power, high-frequency vibration components within the transmitted signal, which may provide discriminant information to detect and recognize the current state of the machine, relevant for fault diagnosis.

SAW-based systems have been previously used to measure slowly changing variables, such as torsion or temperature [3,4]. Nevertheless, SAW systems offer decisive advantages due to their passive working principle and fast interrogation potential. The extent to which these advantages can be used for vibration measurement or condition monitoring on rotating shafts are investigated in this work and verified with a practical measurement setup.

### 1.3. Alternative Technologies

Vibration measurement as such can be accomplished by alternative technologies. Optical systems, like laser vibrometers, can provide extremely high precision on stationary and rotating parts. As an example, measuring with a laser vibrometer opens up great possibilities, as it is very precise and non-cooperative (does not require an additional device on the DUT). However, drawbacks include high system cost due to complex internal signal conditioning, sensitivity to temperature and vibration, measurement of only one single spot per device and high expenditure in the installation of the laser as well as in costs and maintenance. Non-cooperative radio frequency (RF) systems, like Doppler radar, provides lower precision at increased robustness against environmental strain (temperature, vibration, dust), but can suffer from signal fluctuations in the radio channel that might distort the actual measurements. Vibration sensors, like micro-electro-mechanical systems (MEMS) or strain gauges attached to the DUT, are generally available at different kinds of price and quality. Nevertheless, they usually require a power supply and wired data transfer, what may cause difficulties when upgrading existing machines and, in case of battery supply, causes additional maintenance effort.

### 1.4. Structure of the Paper

The structure of this work has been organized as follows. The materials and methods are presented in Section 2, defining, first, the physical principles and signal modeling of the technology proposed. Second, the description of the concept for augmented SAW RFID with external sensor information. Third, the test setup used to evaluate the system is characterized. Fourth, an efficient signal processing implementation in real-time is proposed to evaluate continuously the SAW RFID signal and reconstruct the sensor contributions in a sequential post-processing stage. In Section 3, the concept is implemented with a use-case oriented experimental setup. Continuous measurements using the proposed system are taken from different induction motor shafts, and features are extracted to determine state of the DUT. The presented results are discussed in Section 4. Finally, conclusions and outlook perspectives are considered in Section 5.

## 2. Materials and Methods

Induction motors with rotating shafts are evaluated as "device under test (DUT)" in this work, which generate vibration patterns while operating. The aim is to continuously monitor these patterns in real-time. A piezoelectric vibration sensor is attached to the DUT to measure the vibration signals, converting the mechanical oscillations into electrical signals. The sensor is coupled with a SAW RFID tag, which consists of a reflective delay-line, and it modulates the impedance of the tag with its output. To interrogate the transponder system, including the information of the sensor, a multi-channel frequency-modulated continuous-wave (FMCW) transceiver unit developed at Siemens working at the 2.4 GHz ISM band is used.

Each channel is connected to an antenna element that sends out a signal which is received by a SAW RFID tag with *P* reflectors. The tag antenna is connected to an interdigital transducer (IDT), responsible of converting the interrogation RF signal into acoustic waves, which propagate through a piezoelectric surface. These waves are backpropagated from the reflector structures of the tag to the IDT, converted again into RF energy and sent to the interrogation unit, where the signals are baseband converted and processed. The signal processing takes place, first, in the interrogation unit, where the data dimensionality is reduced, extracting the identification and sensing information. Then, in the post-processing unit, the output information is analyzed.

**Figure 1:**
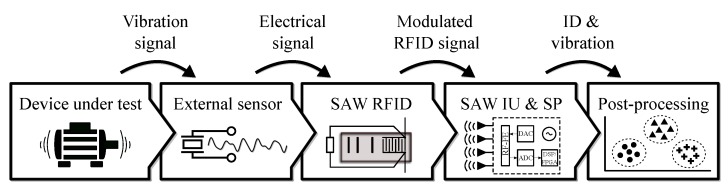
Building blocks of the SAW-based wireless condition monitoring system consisting of: device under test as vibration source, external sensor capturing vibration, SAW RFID tag coupled with the sensor, SAW interrogation unit (IU) and signal processing (SP) stage and, eventually, post-processing stage.

The described building blocks constituting the system are depicted in Figure 1. This modularity offers flexibility for the target application, as individual blocks can be adapted based on specific industrial use-case requirements, as an example, the external sensor used, the interrogation unit architecture or the post-processing implementation.

In this section, first, the theory of SAW RFID technology, represented as the central block in Figure 1, is described. Second, the modulator circuit interface connecting the SAW RFID tag with external sensors and enhancing its sensitivity is explained. Third, the experimental setup with the DUT used as a vibration source to evaluate the system is described and characterized. Fourth, the SAW interrogation unit and signal processing are explained. Finally, the post-processing stage is discussed and defined for the proposed experimental case.

### 2.1. Signal Modeling

Based on the propagation delays defining the transmission model, the physical limits can be calculated and the optimal latency estimated. The signal round trip delay ($\tau_{\mathrm{RT}}$) determines the minimum delay that can be achieved by the system, conditioning the interrogation signal configuration. The $\tau_{\mathrm{RT}}$ results from the electromagnetic wave propagation delay through the air plus the acoustic wave propagation delay on the piezoelectric substrate of the SAW RFID tag, as defined in (1)

(1) $$\tau_{\mathrm{RT}}=\tau_{R}+\tau_{i}=2\left(\frac{R}{c_{0}}+\frac{r_{i}}{c_{\mathrm{SAW}}}\right),$$

where $c_{0}$ is the approximated speed of the electromagnetic waves and $c_{\mathrm{SAW}}$ the speed of the SAW on the piezoelectric substrate, being $c_{\mathrm{SAW}}$ much slower than $c_{0}$[5], what results in $\tau_{i}$ about $10^{5}$ times larger than $\tau_{R}$. These delays define the signal model, carrying identification and sensing information on the transmitted intermediate frequency (IF) signal.

A frequency-modulated continuous-wave (FMCW) interrogation can be implemented with bandwidth $B_{\mathrm{FMCW}}$ and sweep duration $T_{\mathrm{FMCW}}$. The FMCW configuration together with the round trip delay contributions define the signal frequency components $f_{i}$ of the baseband signal, corresponding to the SAW RFID reflectors, as shown in (2)

(2) $$f_{i}=\frac{B_{\mathrm{FMCW}}}{T_{\mathrm{FMCW}}}\cdot \left(\tau_{R}+\tau_{i}\right).$$

Each frequency component $f_{i}$ can be considered as a bit of a code word, while the combination of all components within a signal represent an identification code. The signal phase components $\varphi_{i}$ are also dependent on the delays and the operating interrogation carrier frequency $f_{c}$, as shown in (3)

(3) $$\varphi_{i}=2\pi \cdot f_{c}\cdot \left(\tau_{R}+\tau_{i}\right).$$

Considering multiple transceiver channels, the mathematical model of the resulting beat signal $s_{m}$, on each channel *m*, can be represented as described in (4)

(4) $$s_{m}\left(t\right)=\sum_{i=1}^{P}A_{i}\cdot e^{j(2\pi \cdot f_{i,m}\cdot t+\varphi_{i,m})}+n_{m}\left(t\right),$$

where *P* is the total number of reflectors, which is equivalent to the number of SAW signal frequency components, $A_{i}$ is the amplitude of each reflected signal and $n_{m}\left(t\right)$ represents the measurement noise on each channel.

### 2.2. Augmented SAW RFID Concept for Vibration Measurement

A passive identification and sensing solution for condition monitoring is developed based on an augmented transponder system. Passive solutions are specially suitable for long-term monitoring and predictive maintenance applications, as measurements can be performed independently from battery life limitations.

To capture a complete vibration signature and enable the extraction of discriminant features for condition classification, sensors sensitive to wideband responses are well suited. However, high-frequency components, potentially containing information about the actual state, are expected to vibrate at low power. Consequently, a matching circuit is implemented to enhance modulation sensitivity and capture this information without requiring external amplification. Next, the designed augmented RFID sensing solution is presented.

An advantage of using this modular approach, i.e., sensor coupled externally with the SAW RFID, instead of using an integrated design, is the flexibility to use different sensor solutions to specific vibration spectral ranges of interest, even to other sensing magnitudes, without significant system variations. Hence, using the same interrogation hardware and configuration, even the same SAW RFID tag and modulator circuit concept, various sensors can be matched and evaluated. Furthermore, no mechanical strain is applied to the SAW device. That supposes an enhancement in the lifetime of this approach in comparison to other SAW solutions where the SAW crystal is bended, what can lead to earlier device failure.

#### 2.2.1. Modulation Principle: External Sensor Coupling

The complex termination impedance at the electrical port of the interdigital transducer determines the reflection magnitude and phase of the SAW tag signal [6]. In conventional operation, the surface acoustic waves arriving at the IDT generate a charge distribution and, therefore, an electric voltage between the fingers, reexciting the SAW. If, on top of that, an external source is applied parallel to the IDT or to one or multiple reflectors, acting as a load impedance, the total impedance changes and the reflection behavior is modulated [2] as a function of the corresponding source.

A sensor is coupled to the SAW RFID tag and its contribution is added on top of the transponder signal, which acts as carrier containing the identification code information. As a consequence, the power and phase of the signal change according to the reflection variation. This property can be efficiently analyzed on the interrogation unit in time-domain, by tracking the signal components, i.e., reflected signals, at their characteristic delays.

The extension of the transponder concept as a vibration solution can be suitably completed with piezoelectric accelerometers, among other types of sensors, as they may work without power supply. Their sensing principle is based on an electrical potential generation produced by mechanical vibrations, proportional to the acceleration acting on the sensor material, which is physically described by the piezoelectric effect. The characteristics of the output signal generated by the sensor result in a power correlated with its sensitivity and contain the frequency components of the sensed vibration.

To measure the electric voltage of the sensor, a varactor diode is introduced, working as a capacitive sensor. Thus, the output signal of the sensor modulates the capacitance of the diode and, therefore, the electrical load impedance to the IDT.

The potential dynamic range of the varactor diode based on the sensor modulation can be defined by its equivalent circuit parameters. Commercial models alone may not be enough sensitive to capture all signal components. A solution is here proposed, alternative to active amplification, based on a matching circuit between diode and IDT with a resonant behavior. This modulation principle enables a sensitive passive transponder system.

#### 2.2.2. Circuit Characterization

As the varactor diode changes its capacitance with the voltage signal from the vibration sensor, a voltage-controlled capacitance results. This causes a change in the impedance of the circuit, leading to a change in the impedance of the interdigital transducer. The vibration signal acts through the circuit directly on the impedance of the transducer and, thus, on the reflected signal.

Therefore, a resonant circuit is designed to achieve larger power and phase changes of the reflectivity (impedance) without high signal loss, maintaining the signal-to-noise ratio of the SAW RFID without significantly reducing the reading range of the tag. The transfer function of the circuit enhances the modulation sweep and, thereby, increases the sensitivity of the system, rising low-energy signal components from the noise floor.

**Figure 2:**
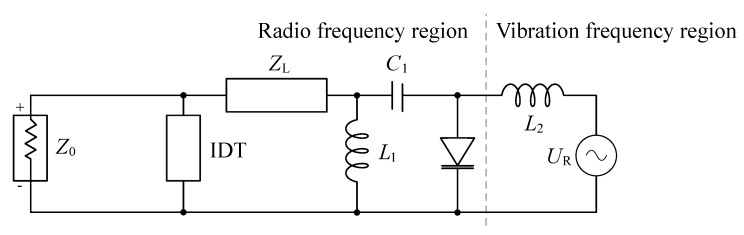
Equivalent modulator circuit of the passive vibration and identification transponder concept represented with lumped elements. Mechanical vibration and radio frequency range circuit sections are separated by the gray dashed line.

The proposed circuit, represented in Figure 2, can be analyzed in two sections, corresponding to the radio and vibration frequency regions. An inductance ($L_{2}$) is placed in the boundary between the two regions, working as a first-order low-pass filter to decouple RF signals from the modulator sensor output ($U_{R}$), represented as a voltage source, and the RF section.

On the RF section, the varactor diode is connected in parallel. The resonant circuit is formed by a capacitor in series ($C_{1}$) and an inductor in parallel ($L_{1}$). A transmission line ($Z_{L}$) is located between diode and IDT, matching the phase of the signal to optimize the modulation sweep. Finally, the port impedance ($Z_{0}$) is depicted in parallel to the IDT.

To enhance the sensitivity of the measurement system, the best combination of $L_{1}$ and $C_{1}$ for the tuning components are selected to obtain a resonance frequency close to the interrogation carrier frequency. The depth of the resonance function plays also a significant role. Depending on how steep the function fall within the interrogation bandwidth $B_{\mathrm{FMCW}}$ is, the modulation sensitivity changes. Since reflectivity becomes a function of the sensor voltage, the design criteria is based on optimizing the variance throughout the dynamic range of the sensor (5)

(5) $$\frac{d\Delta \Gamma_{\mathrm{IDT}}}{dU_{\mathrm{sens}.}}=0\Rightarrow \underset{U\in [U_{\min.},U_{\max.}]}{arg max}\;\Delta \Gamma_{\mathrm{IDT}}\left(U\right),$$

where $\Gamma_{\mathrm{IDT}}$ corresponds to the reflectivity of the IDT, dependent on the modulation voltage $U_{\mathrm{sens}.}$. To approach this optimization problem practically, the external sensor is previously characterized. The piezoelectric sensor response was measured for vibrations up to 9 kHz, although it has the potential to detect even higher frequencies. A vibration at 1 G corresponds to an output voltage of ≈±100 mV with $U_{\mathrm{bias}}=0$ V. This reference is used to evaluate the performance of the circuit.

**Figure 3:**
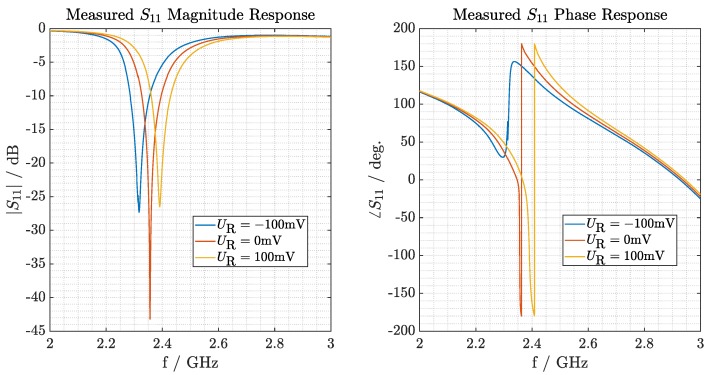
Measured $S_{11}$ parameters of the modulation circuit with reverse voltage states ($U_{R}$) equal to −100, 0 and 100 mV, which correspond to the reflected magnitude (**left**) and phase (**right**). The interrogation frequency modulation might sweep between 2.4 and 2.4835 GHz.

The output voltage of the sensor modulates the capacitance of the varactor diode and this, in turn, shifts the resonant function of the circuit. Figure 3 presents the relationship between the magnitude and phase of the reflectivity (left and right plots, respectively) and the voltage states $U_{R}\in \left\{-100,0,100\right\}$ mV of a controlled voltage source, depicted with three different curves.

As design limitations for practical implementation, the following conditions at the modulation voltage limits are defined. At the reference frequency f=2.45 GHz, a maximum magnitude attenuation of −3 dB when $U_{R}=-100$ mV is stipulated, as higher attenuation may result in an impractical reading range. Setting this condition, at $U_{R}=100$ mV the magnitude of the reflected signal decreases to nearly –8 dB. This SNR reduction affects both the power and phase variability of the reflected signal along the complete measurement range, nonetheless, maintaining a reading range of R>50 cm. On the other hand, the phase modulation has a variation range of $\Delta \varphi \approx 30^{\circ }$. With the characterized performance, the vibration information of interest contained in the backscattered signal of the SAW RFID tag can be demodulated in the interrogation unit.

### 2.3. Experimental Setup

A measurement setup consisting of three induction motors with different conditions is evaluated to verify the performance of the proposed identification and sensing system. The vibration signatures corresponding to each of the different motor states are analyzed. One of the induction motors has a damaged front bearing, labelled in the following as a faulty condition. The other motors work under regular operation, labelled in the following as a healthy condition. A cylindrical printed circuit board is clamped on the motor shafts, including the augmented SAW RFID circuit and four patch antennas with equidistant separation, covering $360^{\circ }$, what allows for continuous measurement around the shaft. A COTS piezoelectric sensor is attached to the motor shaft and coupled with the transponder. Four *Huber+Suhner* antennas with 9 dBi gain are equally spaced from the motor shaft around its center. Each antenna is connected to an independent interrogation unit channel, operating simultaneously as parallel transceivers. The signals are processed in the interrogation unit and the output is sent to a computer system, where the post-processing implementation takes place.

**Figure 4:**
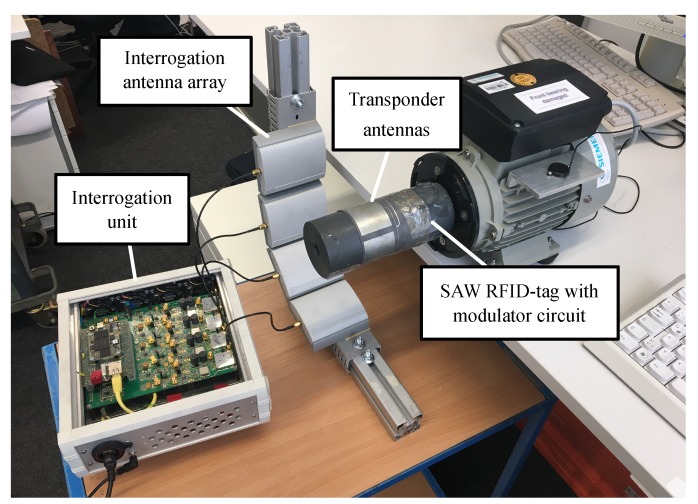
Photo of the experimental setup used to evaluate vibration measurements on motor shafts.

The experimental setup is shown in Figure 4. The designed transponder is clamped on the motor shaft and it is located in the center of the interrogation antenna array. The array is fixed on an aluminium frame fixed on a separated platform, decoupling the antenna structures from motor vibrations.

The rotation frequency of the motor shaft corresponds to 5940 rpm during operation without load, with single-phase AC motors using a 50 Hz power line. The resulting 1% loss corresponds to the speed difference between the stator and rotor, creating the torque of the motor. Thus, the strongest component in the oscillation spectrum is expected to be around 100 Hz. With a load in the engine, a higher loss of speed between 1 and 10% occurs.

A reference system is analogously used to first evaluate the experimental setup independently from the modulation circuit and SAW RFID carrier demodulation contributions to the signals. Hence, sensor output signals are exclusively evaluated, which are expected to be highly correlated with the signals obtained using the developed SAW RFID system in this work.

As a reference evaluation, the vibration sensors are coupled on the motor cases and connected with a shielded cable to an oscilloscope, minimizing external electromagnetic influences. Consequently, the influence of the transfer function from the augmented RFID concept in addition to the interrogation system and channel or environmental influences are left aside. With this procedure, vibration spectral components present on the motors are initially identified.

Ultimately, to derive the actual vibration (ground truth), the transfer function should be considered and the corresponding normalization applied. The contribution of the sensor transfer function is, however, equal for both reference and evaluated systems, thus, this task falls outside the system characterization purposes of this work.

**Figure 5:**
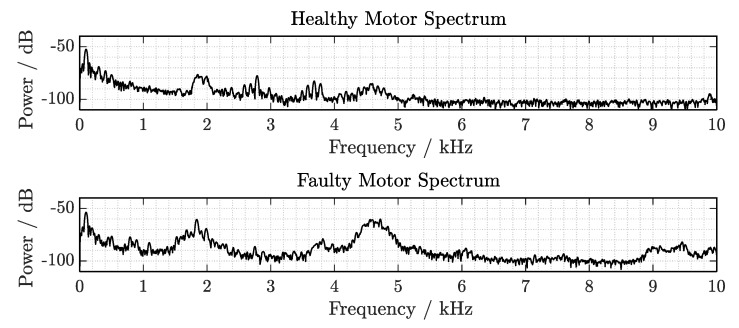
Spectrum of the induction motors measured on their casing with the vibration sensor connected to an oscilloscope, using a sampling rate of $f_{s}=20$ kHz.

Figure 5 shows the spectra of the recorded signals with the reference system. The measured devices under test were a motor with healthy bearings (upper plot) and, in comparison, a motor in which the front motor bearing is damaged (lower plot). These measurements on their casing show the vibration spectrum with the particular contribution of the sensor transfer function response. Both spectra are recorded with a sampling frequency of 20 kHz, in order to later evaluate additional effects from signal undersampling.

The signal components for each operating condition up to the frequency limit $f_{s}/2=10$ kHz are observed. The main components of the healthy motor spectrum can be identified around 100 Hz ($f_{\mathrm{main}}=2\cdot f_{\mathrm{AC}}$) with an SNR of about 50 dB, corresponding to the main vibration frequency generated by the motor shaft rotation, in addition to its harmonics. Further frequency components on the kilohertz range occur in particular from 1.8 to 2 kHz, at 2.78 kHz, from 3.5 to 3.8 kHz and from 4.4 to 4.8 kHz, with lower energy content.

On the other hand, on the faulty condition signature, significant wideband changes can be appreciated in the higher frequency range. In the range from 750 to 900 Hz, frequency components, which were not present in the healthy condition, are observed. In addition to the higher energy contributions at the initial spectral range covering a wider band from 1.5 to 2 kHz, more contributions are present from 4.3 to 5 kHz and some components up to 9 kHz.

### 2.4. Multi-Channel Interrogation Concept and Real-Time Signal Processing Implementation

Taking advantage of the low-latency working principle of the transponder readout, real-time signal processing is aimed to be efficiently designed in order to achieve high measurement rates. Besides, the interrogation unit has the challenge of taking measurements while the target is rotating several times per second, since the tag is clamped on the shaft to be monitored. The sensing solution described in Section 2.2, shows that sensor information is modulated on the power and phase of the SAW RFID signal. During DUT operation, the signal is additionally modulated by the shaft rotation, since the cylindrical antenna radiation pattern is not strictly isotropic.

Depending on the radiation pattern of the cylindrical antenna and the power of the sensor output signal, a single-channel interrogation approach fails when trying to extract low-energy vibration components (such as high-frequency vibrations) from the spectrum, due to a reduction of the system sensitivity and the emergence of additional components induced by the transponder rotation, hiding sensor signal components potentially relevant for condition analysis. Thus, an antenna configuration is proposed to compensate the influences on the signal contribution added by the shaft rotation and to enable the acquisition of relevant features for condition monitoring algorithms.

#### 2.4.1. Signal Processing

The interrogation unit is responsible for the signal acquisition and processing tasks. A parallel four-channel frequency-modulated continuous-wave (FMCW) interrogation is considered. Each FMCW ramp has a duration of $T_{\mathrm{FMCW}}=100$ μs and a bandwidth of $B_{\mathrm{FMCW}}=83.5$ MHz. This configuration offers a sensing sampling rate of $1/T_{\mathrm{FMCW}}=10$ kHz, since single sensor signal samples are extracted from each interrogation measurement. Furthermore, the reflector delay resolution is determined by the bandwidth, i.e., the ability to distinguish two or more reflected signals with different delays. Using the described interrogation configuration, since the resolution is equivalent to $1/(2\cdot B_{\mathrm{FMCW}})\approx 6$ ns, expected reflector signals of conventional COTS SAW RFID tag signals can be unambiguously evaluated.

**Figure 6:**
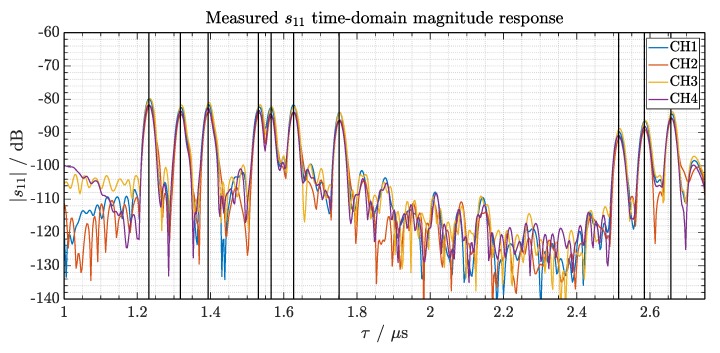
Measured SAW-tag response with the four interrogation channels superimposed. The interrogation ramp duration determines the measurement rate of the SAW RFID signal, while the bandwidth determines the physical resolution. The start bit of the coding scheme arises at 1.22 μs and the stop bit is observed at 2.64 μs. The fourth and fifth reflector peaks are located close to each other, resulting in a delay difference of $\Delta \tau_{4,5}=32$ ns, what agrees with the configuration limits for unambiguous processing.

Based on the signal processing concept proposed in [1], the IF signal acquired on each interrogation is transformed into time-domain using an inverse fast Fourier transform. The spectral components corresponding to the SAW RFID signal are detected with a peak search algorithm and an adaptive noise threshold, designed with prior knowledge of the delay-line characteristics. Figure 6 shows a measured SAW-tag response after the transformation, with the output from the four interrogation channels superimposed.

The combination of signal delays (peak positions) determines the identification code, while their power and phase contain the motion and sensor contributions from the approach described in Section 2.2. Consequently, within a ramp duration, an algorithm is responsible of transforming the raw data into spectrum, search the signal components and store their delay, power and phase in order to not loose information. Once the response signal is detected, spectrum decoding follows [7,8].

The identification code is a static parameter. Considering the latter, averaging can be implemented to reduce the noise floor and, thus, the decoding error rate. This approach can also be used for slow varying physical magnitudes, such as temperature or pressure [9,10]. Nevertheless, vibration is a high dynamically changing phenomenon, with significant information transmitted in its oscillation rate, what demands a system approach able to acquire and process its information at a higher sampling rate in order to unambiguously capture all its components. Therefore, an efficient signal decoding and demodulation in real-time enables the system to achieve this aim. The properties of the proposed transponder approach become very suitable, as low-latency transmission and reception of signals can be established to transmit the signals continuously, with a master latency mainly defined by the signal propagation delay.

Model-based approaches [11,12] are an alternative for signal processing which can outperform the accuracy and resolution achieved by the fast Fourier transform (FFT). These type of algorithms can be implemented using previous knowledge of the coding scheme as an advantage. However, to use them for vibration sensing, efficient implementation of operations, such as matrix inversion, is required in order to continuously process the signals in real-time.

**Figure 7:**
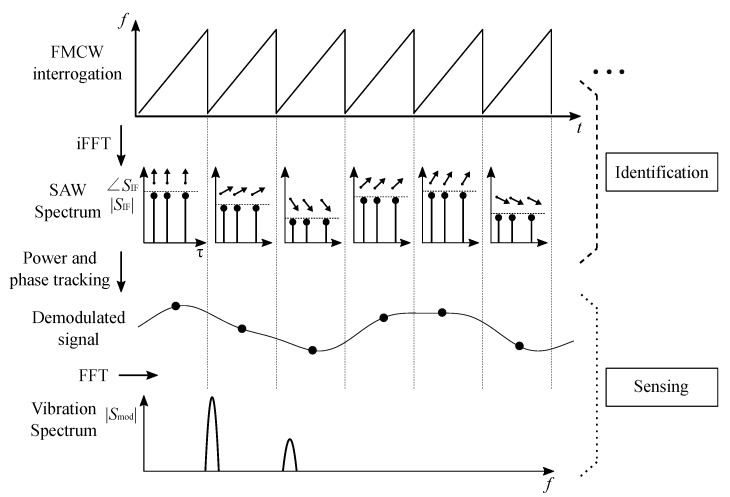
The interrogation ramp duration determines the measurement rate of the SAW RFID signal and, therefore, the sensing sampling rate, which is represented in the first plot. The reflected signal components (peak positions), represented in the second plot, determine the identification code while the power and phase tracking, represented in the third plot, extract the signal components from its spectrum. Successive interrogation ramps retrieve the sensor (vibration) signal, which is transformed block-wise to extract its signal components in frequency-domain, as represented in the lower plot.

Vibration signals contain information within their periodicity, so transformation into frequency domain usually can reveal significant information about the source, by decomposing the vibration signal into its frequencies and their corresponding energy. Hence, a second FFT is proposed to reveal vibration frequencies within a measured time window. Frequency patterns may indicate the nature of the problem, such as shaft misalignment or damaged bearings, while the power of each frequency may reveal the severity of the problem. A summary of the described process is depicted in Figure 7.

#### 2.4.2. Antenna Array Configuration

As characterized by measuring the cylindrical transponder antenna radiation pattern, the shaft rotation introduces additional frequency components in the modulation spectrum, potentially hiding sensor signal components of interest. Hence, a particular antenna array configuration is conceived to compensate the modulation introduced by the anisotropic radiation pattern. The proposed approach is implemented with a simple online operation in the processing unit, without delaying the signal processing time window over the ramp duration limit.

**Figure 8:**
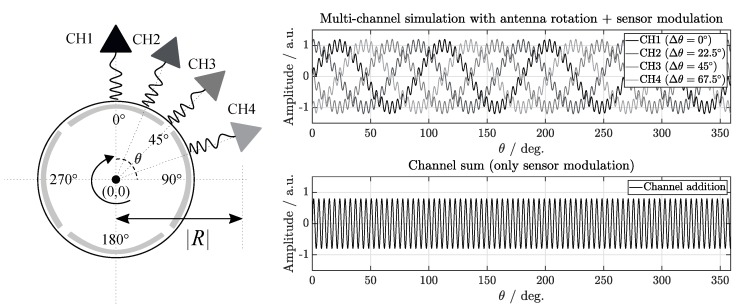
Proposed antenna configuration to compensate the signal contribution of the shaft rotation (**left**). Four equidistant patches at the cylindrical transponder antenna covering the shaft receive and reflect the interrogation signals back with a specific delay, power and phase given by the SAW RFID tag coupled with the sensor. The in-phase components corresponding to the sensor signal are added up, while the out-of-phase components corresponding to the motion are subtracted (**right**).

All FMCW interrogation channels are synchronized to transmit and acquire the signals simultaneously. The antenna rotation generates a periodic pattern which comprises $N_{r}=4$ modulation cycles over a complete shaft rotation. Thus, an antenna array is configured, successively shifting the location of each antenna element by $\Delta \theta =2\pi /(M\cdot N_{r})=\pi /8$ with respect to the motor shaft center at the same radial distance $\left|R\right|$, as shown in Figure 8 (left). This causes a destructive signal superposition when adding up the received signals. While the transponder antenna rotation pattern is dependent on the orientation angle of the shaft with respect to the interrogation antenna, the sensor contribution to the carrier signal is independent of this variable. Hence, the rotation interference is cancelled and the sensing signal-to-noise ratio is enhanced.

The number of interrogation antennas (*M*) can be reduced, nevertheless, also reducing the SNR of the signal and, therefore, reducing the sensitivity of the demodulation approach. Due to the low energy of the discriminant modulation signal components for condition monitoring and the fact that a passive solution under harsh conditions is used, the results using M=4 antennas are shown in this work, i.e., using all channels of the interrogation system.

As a result, four phase shifted replicas of the antenna rotation signal are obtained in the processing unit, while the sensor modulation is assumed to be phase-synchronized. Thus, adding the signals measured with the different channels leads to a cancellation of the antenna modulation influence (destructive overlap) and the sensor modulation contributions are added (constructive overlap), as simulated in Figure 8 (right). Mathematically, each *m* antenna element $\left(r_{x},r_{y}\right)$ location with respect to the shaft center can be calculated, as shown in (6a) and (6b)

(6a) $$r_{x,m}=\left|R\right|\cdot \cos\left(2\pi \frac{m}{M\cdot N_{r}}\right)$$

(6b) $$r_{y,m}=\left|R\right|\cdot \sin\left(2\pi \frac{m}{M\cdot N_{r}}\right).$$

The proposed antenna array configuration measures M=4 rotation signals with a modulation phase shift $\varphi_{r,m}=\varphi_{r}+m\cdot \pi /2$, where $m=\left\{0,\ldots ,M-1\right\}$. By simultaneously measuring the SAW RFID signals, the rotation contribution is compensated. The resulting signal model can be described as

(7) $$s_{\mathrm{mod}.}=\sum_{m=0}^{M-1}e^{j(2\pi f_{r}+\varphi_{r}+m\cdot \pi /2)}+e^{j(2\pi f_{\mathrm{sensor}}+\varphi_{\mathrm{sensor}})}=M\cdot e^{j(2\pi f_{\mathrm{sensor}}+\varphi_{\mathrm{sensor}})}.$$

### 2.5. Post-Processing

The vibration spectrum of rotating motors may contain frequency components revealing unambiguous information about the motor condition, as shown in the experimental setup (Section 2.3). Besides, changes on vibration frequency patterns over time may reveal unique signatures and tendencies for early fault detection.

To better understand the nature of the measured vibration signals and to allow the investigation of trends [13], a post-processing is proposed not exclusively based on frequency domain. So, a time–frequency analysis is implemented to extract continuous information about the vibration signals and estimate the time evolution of the vibration frequency content. Hence, time-variant condition-related features can be extracted for fault diagnosis, allowing to correlate the dependency between properties of vibration signatures and conditions. In addition, this type of continuous analysis allows temporal localization of particular frequency components, eventually becoming present on vibration signals, and their succession order for potential predictive modelling.

In the post-processing implementation, a time window is shifted over time, slicing the retrieved time-domain vibration signals into vectors of size *N* with partial overlap. Within the resulting (relative) short-time duration windows, the characteristics of the segmented vibration signals do not change significantly, so, a semi-stationarity assumption can be made within the selected time window. The vibration frequency components of each vector are calculated and concatenated iteratively, forming a time–frequency matrix. The resulting matrix contains the local vibration spectra recorded with time and frequency indices, what can be written as

(8) $$\begin{array}{cc} X[k,i]= & \sum_{n=0}^{N-1}x\left[n+i\cdot D\right]\cdot w\left[n\right]\cdot W_{N}^{k\cdot n}\\ \overset{n\to n-i\cdot D}{=} & \sum_{n=i\cdot D}^{N-1+i\cdot D}x\left[n\right]\cdot w\left[n-i\cdot D\right]\cdot W_{N}^{k(n-i\cdot D)}\;\mathrm{for}k=0,1,\ldots ,N-1, \end{array}$$

where the window sequence w[n] of length *N* is used to select a finite-length (local) segment from the sliding sequence x[n+i·D] and reduce the spectral leakage. Each segment is overlapped with its adjacent block by $N_{\mathrm{ov}.}=N-D$ samples and the frequency and time denoted by the frequency index *k* and time index *i* of X[k,i] are defined in Equation (9)

(9) $$f_{k}=\frac{k}{N\cdot T}=\frac{k}{N}\cdot f_{s}$$

and Equation (10), respectively,

(10) $$t_{i}=\left(\frac{N}{2}+i\cdot M\right)\cdot T,$$

where $t_{i}$ is set to the center of each sliding window. Consequently, the time stamp of the local spectrum can be obtained and, thus, events may be identified and temporally localized, also enabling the examination of the time evolution of the vibration spectra.

The processing block size (*N*) can be chosen depending on the type of vibration characteristics aimed to be extracted. If the choice of *N* is inappropriately large, vibration transients or fast energy variations, e.g., impact of hammering against floor, may not be resolved. So, a compromise between simultaneous time and frequency resolution must be found, analogous to the Heisenberg uncertainty principle. For this work, a window length of N=512 samples (i.e., 51.2 ms) was programmed with 90% overlap. Nevertheless, the flexibility of the system allows to adapt the length *N* according to different use case requirements. Alternatively, other time–frequency analysis methods for machinery fault diagnosis with different time–frequency resolution dependencies can be used [14].

From the resulting transformation, different statistical features can be then extracted to identify the differences between vibration signal patterns [15]. Evaluating the proposed experimental setup, common as well as discriminant frequency components are observed between condition types, which were persistent over measurement repetitions. With the aim of automatically recognize the state of the device under test, a model-based classification algorithm has been efficiently implemented based on distance calculation from each new measurement transformed into a feature space to the center of each defined cluster class, as shown, next, in the results section. The states of the device under test (classes) were defined according to a use-case classification criteria between healthy and faulty conditions, in addition to a non-operating state.

## 3. Results

The results obtained using the building blocks, defined and explained in materials and methods, are presented in this section. First, the anisotropic radiation pattern of the reflected signal by the cylindrical transponder antenna is characterized and the results between uncompensated and compensated processing are compared. Second, the reconstructed vibration spectra measured directly on different motor shafts using the proposed system are analyzed. Finally, the condition clustering performance is evaluated on a three-dimensional feature space, where distinctive discriminant cluster decision surfaces are defined. These results demonstrate the potential of the system for condition monitoring applications on rotating shafts.

### 3.1. Compensation of Shaft Rotation

**Figure 9:**
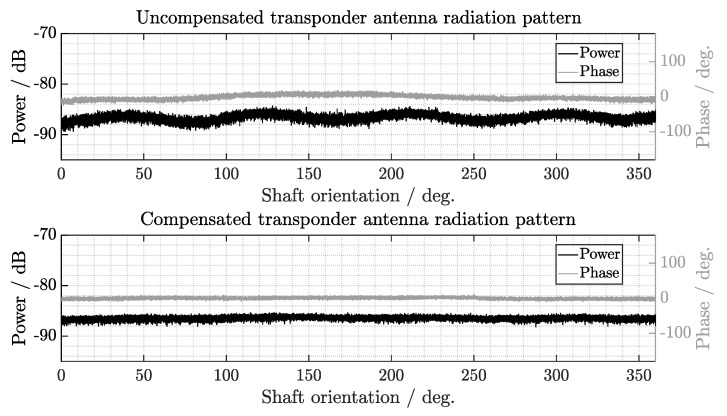
Characterization of the transponder antenna radiation pattern over 360${}^{\circ }$ shaft orientation. The reflection signal was continuously measured during shaft rotation at low frequency with an uncompensated single channel approach (**above**) and with a compensated multi-channel approach (**below**).

Considering the motor shaft rotation frequency $f_{\mathrm{main}}=100$ Hz, the ramp duration $T_{\mathrm{FMCW}}=100$ μs enables 100 measurements over a single rotation ($1/(T_{\mathrm{FMCW}}\cdot f_{\mathrm{main}})$). Due to the anisotropic radiation pattern (without notch angles) a background periodic modulation of the SAW-tag carrier signal is generated by the shaft rotation. This contribution is dependent on the orientation angle of the shaft with respect to the interrogation antenna, as it can be observed in Figure 9.

With the methodology described in Section 2.4, the rotation is compensated when measuring the signals on the motor shaft using the multi-channel approach depicted in Figure 8. Besides the cancellation of the background anisotropic modulation pattern generated by the cylindrical transponder antenna rotation, a reduction of the standard deviation between the uncompensated and compensated spectra can be observed after the signal averaging. As a result, the sensitivity of the demodulation processing is enhanced, leading to an improvement of the vibration signal reconstruction and an enhancement of the condition monitoring performance.

### 3.2. Spectral Signatures

Blocks of N=512 consecutive vibration data samples are continuously measured with a sampling rate of $1/T_{\mathrm{FMCW}}=10$ kHz and sequentially processed from the extracted SAW RFID reflected signals. This vibration information sensed by the external sensor is modulated on top of the ID. The vibration signals are then transformed into frequency-domain using a fast Fourier transform and their frequency components are unveiled in spectral representation for further analysis.

**Figure 10:**
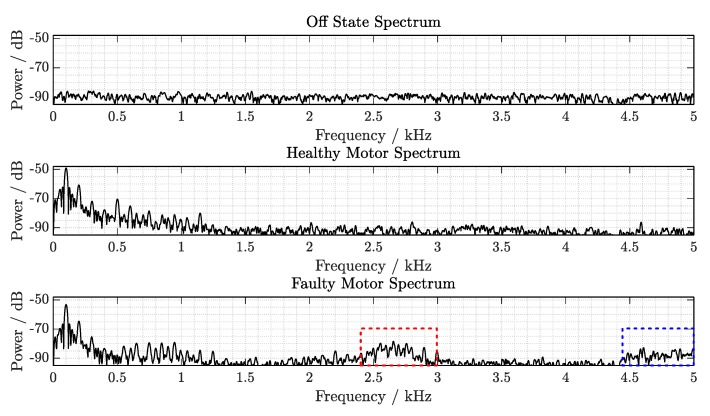
Demodulated vibration spectrum from different motor conditions: off (**above**), healthy (**middle**) and faulty (**below**). In the healthy and faulty spectra, the rotation frequency and its harmonics are visible, while additional information characteristics corresponding to the particular conditions arise at higher frequencies. The grid division of the plot is set at 100 Hz/5 dB to ease the reading.

In Figure 10, three different spectral signatures are shown, corresponding to three motor conditions. In the first subfigure (above), the spectrum depicted corresponds to the off state, the condition defined when the motor is not operating. Thus, no predominant spectral component arises. The second subfigure (middle) corresponds to the healthy operating state, representing the correct motor operation condition, as the motor has not been modified. The third subfigure (below) corresponds to the faulty operating state, indicating that there is a defect in the motor operation. The resulting signals under this condition contain an additional wideband contribution in the spectrum.

In the healthy and faulty motor spectra, the strongest frequency component can be identified around 100 Hz, corresponding to the main rotation frequency of the motor shaft. Around 200, 300 and 400 Hz, a first, second and third harmonics of the rotation frequency follow, respectively. At 500 Hz, a spectral component arises only in the healthy condition spectrum, with its corresponding harmonics. Between 600 Hz and 1.2 kHz, different spectral components arise in both healthy and faulty conditions. Besides, in the lower subfigure, wideband signals arise within two spectral regions from 2.4 to 3 kHz and from 4.4 to 5 kHz, which are discriminative for the faulty motor condition.

### 3.3. Condition Clustering

The measured vibration signatures show features containing the information about the state of the motors. Using features makes the condition monitoring process more efficient, as less computational power is required to classify the motor condition in real-time.

Thus, different features are extracted to represent the state of the vibration source. The energy of the main frequency component, i.e., rotation frequency, is extracted as an indicator of motor operation, which defines the cluster separation between off and operating states. Furthermore, it may indicate a deviation of the rotation frequency, e.g., when the motor rotation frequency decreases.

Additional features can be extracted to classify the different operating conditions. Some of them, may be more relevant to evaluate a temporal evolution. In order to use robust characteristics along extended measurements and to decrease the large scattering of features extracted from high-frequency components for clustering, the average spectrum was calculated over specific spectral frequency intervals of interest.

As described in Section 2.3, three motors are used to generate the datasets. Features obtained from measurements of each motor are extracted and analyzed. One motor has been characterized as faulty, while the other two have not been modified, which are categorized as healthy.

**Figure 11:**
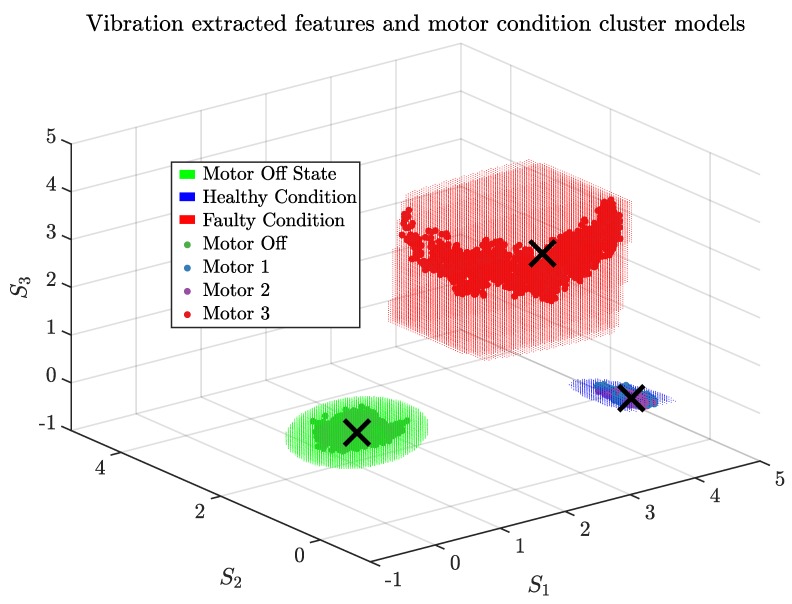
Condition classification results after feature extraction. The vibration extracted features and motor condition cluster decision surfaces are visualized in a three-dimensional space. The first feature ($S_{1}$) represents the spectral energy of the rotation frequency component ($f_{\mathrm{main}}=100$ Hz), the second feature, ($S_{2}$), represents the average energy between 2.4 and 3 kHz, and the third feature, ($S_{3}$), represents the average energy between 4.4 and 5 kHz. The crossmarks represent the center of the clusters, the bigger dots depict the features extracted from the measurements and the smaller dots represent the cluster boundaries.

Figure 11 shows the clustering of three conditions (off, healthy and faulty) from four different datasets (motor off and motors 1, 2 and 3). All measurements have been taken using the same measurement setup, shown in Figure 4, which consisted of the motor shaft with the cylindrical antenna connected to the SAW RFID coupled with the external vibration sensor through the described modulator circuit of Section 2.2. Thus, variance introduced by component tolerances or frequency weighting affected by different signal transfer functions between datasets are discarded.

Three distinctly separated clusters can be defined using the proposed algorithm with centroids spaced by significant Euclidean distances without overlap between cluster decision surfaces in the depicted three-dimensional feature space.

## 4. Discussion

The results have shown the potential of the proposed system for real-time condition monitoring applications on rotating motor shafts. The demodulated vibration measurements have demonstrated that discriminant features can be extracted and clustered according to the motor conditions. High-frequency components at different spectral regions have been essential to identify the healthy and faulty motor states.

### 4.1. Interrogation Unit Configuration

A fast interrogation rate at $1/T_{\mathrm{FMCW}}=10$ kHz was required to obtain a wide spectral characterization. This has been possible due to direct wireless sensing without communication protocols, as only the propagation paths determine the master delay of the system. A similar FMCW SAW reader concept for temperature sensing on high-speed, high-voltage motors, implementing the same ramp duration, was introduced in [16], and compared with other continuous wave modulation approaches (FSCW and S-FSCW) in [17].

At the same time, a minimum bandwidth was also necessary to separate the different reflector signals. Hence, a frequency synthesis able to achieve a specific $\mu =B_{\mathrm{FMCW}}/T_{\mathrm{FMCW}}$ ratio was necessary, where a high resolution ($B_{\mathrm{FMCW}}$ maximized) with short ramp configuration ($T_{\mathrm{FMCW}}$ minimized) is suitable. Nevertheless, the higher the ratio μ, the further the spectral components of the SAW RFID signals are shifted in frequency. This condition adds into consideration the sampling rate of the analog-to-digital converter, which will determine the highest frequency component, i.e., largest signal delay, that can be sampled and, hence, the maximum ratio μ that can be implemented.

### 4.2. Transponder Signal Processing and Motion Compensation

The designed matching circuit between RFID and sensor has been fundamental to sense the discriminant vibration features, since it has enabled a high sensitive modulation on top of the identification signal. Low amplitudes corresponding to high-frequency vibrations were acquired. As a result, a wireless solution dismissing power supply was achieved.

To capture the augmented RFID information at the frequency rate given by the FMCW ramp duration of the interrogation unit, a real-time signal processing implementation is required to transform the received data to time-domain, where the signal components can be efficiently extracted given the coding scheme of the SAW RFID [8], containing the identification code on their pulse delays and the sensing information on their power and phase. Besides, a successive post-processing step allows a flexible data analysis which can be adapted to specific use case requirements.

Additionally, given the special characteristics of the interrogation unit with a M=4 channel parallel architecture, compensation of rotation was feasible using a particular antenna array configuration. By measuring vibration with the designed transponder antenna while the shaft is rotating, a strong modulation component due to the anisotropic radiation pattern is measured and, as a consequence, the sensing accuracy is reduced. This has been compensated on the interrogation unit, cancelling the background modulation generated by the shaft rotation, while the sensor contributions were added constructively.

### 4.3. Sensor Mounting on Shaft

There is a difficulty when designing a practical measurement setup which can be mounted on the motor shaft with minimum effort. Retrofittability or adaptation to existing machines is very important. The proposed small-size, analog transponder system makes the mass of the sensor and SAW-tags negligible, in relation to the centrifugal force of the motor. Milling the shaft may be discarded due to the potential of causing a structural damage to the shaft. Gluing or sticking the transponder may not be reliable, as adhesive strength may be reduced over time due to the centrifugal force when the shaft is rotating or when operating at high temperatures. Thus, an intrinsically safe installation method has been proposed, with sensor signal readability around the shaft using a cylindrical circuit board with four patch antennas. The transponder has been clamped around the shaft, with the advantage of being screwless, without damaging the system and avoiding additional processes during production, so the sensor system is not required to be mounted in advance.

Apart from the shaft rotation, additional physical condition variations modulating the transponder substrate, such as temperature or pressure fluctuations, may also affect the processed signals. However, while those influences fluctuate slower, they can be discriminated in frequency domain from the contributions generated by DUT vibrations, which are expected to fluctuate faster. As shown in the results, the frequency ranges used for classification between healthy and faulty motor states have started around 2 kHz.

### 4.4. Comparison with Alternative Systems

The wireless direct reading without digital communication protocols has several advantages. Since the reader is capable of reading multiple sensors simultaneously, the number of sensors can be easily increased without further, potentially costly, hardware modifications. Given a suitable alignment of antennas, a single reader can be used to illuminate a huge number of low-cost sensors, making the implementation very cost-effective when multiple monitoring spots are required.

Alternative solutions using SAW technology, such as SAW resonators with integrated vibration sensing capability and combined with time domain reader methods [18], present their advantages, achieving high measurement update rates [19,20,21]. Nevertheless, such approaches are usually fabricated on rigid piezoelectric materials and, as such, have a higher risk of breaking due to stress overload (as their design must combine proper acoustic wave propagation properties and, furthermore, be sensitive to vibrations within a specific frequency range). In some cases, however, this procedure may make the SAW sensor itself more fragile and lead to earlier replacement. Besides, specific geometrical designs must be intended to meet certain vibration ranges, therefore, previous specifications of the use-case are required before manufacturing, reducing the flexibility of the development cycle.

## 5. Conclusions

Real-time vibration measurements on rotating motor shafts have been achieved using the proposed system based on SAW RFID tags coupled with sensors. A complete system solution for condition monitoring has been evaluated in a use-case oriented experimental setup. Compared to existing solutions, the transponder system can be installed directly at the location of interest, as the sensing device is wireless and designed to be fully passive, i.e., without the need for power supply, battery or wiring. Therefore, phenomena such as strain and vibrations can be measured at more appropriate locations. The high measurement speed enables a true direct measurement of vibration frequencies in the kilohertz region, until 5 kHz. As the design incorporates an external sensor for acquisition of vibration information, the system can be easily tailored to various ranges of applications, i.e., different vibration frequency bands and sensitivities, which can be implemented with the corresponding modifications using the same system concept.

As an outlook, this condition monitoring system can be further developed in combination with advanced algorithmic approaches, such as deep learning for predictive maintenance [22,23]. This contribution can represent an added value in the building blocks towards future smart factories for industrial sensing and automation. Expertise in specific use-cases can lead to extraction of additional features and thus, improve the performance. Furthermore, regression models could be implemented to identify the state of deterioration and the expected lifetime of the DUT.
